# Inverse Associations between Obesity Indicators and Thymic T-Cell Production Levels in Aging Atomic-Bomb Survivors

**DOI:** 10.1371/journal.pone.0091985

**Published:** 2014-03-20

**Authors:** Kengo Yoshida, Eiji Nakashima, Yoshiko Kubo, Mika Yamaoka, Junko Kajimura, Seishi Kyoizumi, Tomonori Hayashi, Waka Ohishi, Yoichiro Kusunoki

**Affiliations:** 1 Department of Radiobiology/Molecular Epidemiology, Radiation Effects Research Foundation, Hiroshima, Japan; 2 Department of Statistics, Radiation Effects Research Foundation, Hiroshima, Japan; 3 Department of Clinical Studies, Radiation Effects Research Foundation, Hiroshima, Japan; University of Nebraska Medical center, United States of America

## Abstract

Reduction of the naive T-cell population represents a deteriorating state in the immune system that occurs with advancing age. In animal model studies, obesity compromises the T-cell immune system as a result of enhanced adipogenesis in primary lymphoid organs and systemic inflammation. In this study, to test the hypothesis that obesity may contribute to the aging of human T-cell immunity, a thousand atomic-bomb survivors were examined for obesity status and ability to produce naive T cells, i.e., T-cell receptor excision circle (TREC) numbers in CD4 and CD8 T cells. The number of TRECs showed a strong positive correlation with naive T cell numbers, and lower TREC numbers were associated with higher age. We found that the TREC number was inversely associated with levels of obesity indicators (BMI, hemoglobin A1c) and serum CRP levels. Development of type-2 diabetes and fatty liver was also associated with lower TREC numbers. This population study suggests that obesity with enhanced inflammation is involved in aging of the human T-cell immune system. Given the fact that obesity increases the risk of numerous age-related diseases, attenuated immune competence is a possible mechanistic link between obesity and disease development among the elderly.

## Introduction

Advancing age is accompanied by a variety of alterations in the immune system. These alterations are most prominently recognized in T-cell immunity, i.e., involution of the thymus and diminished T-cell production and functions [1]. Accumulating evidence suggests that such aging of the T-cell immune system may accelerate under obese conditions [2]. It has been known that *ob*/*ob* and *db*/*db* mice, rodent models of obesity, display markedly involuted thymuses and have significant defects in T cell responsiveness [3], [4]. Mouse model studies further suggest that obesity compromises T-cell production through an increased number of adipocytes and their secreted cytokines within the lymphoid microenvironment of the thymus and bone marrow [5], [6]. Moreover, systemic proinflammatory cytokines that are likely derived from adipose tissue may enhance myeloid skewing of hematopoietic stem cells, leading to fewer lymphoid progenitors [7], on which thymic T-cell development depends. Although preceding studies, typically on leptin and growth hormone, have provided important insight into the relationship between obesity and T-cell aging in humans [8]–[11], the effects of obesity indicators and related diseases on human thymic T-cell production need to be examined in a cohort-based study. Our work with the hypothesis that obesity contributes to a reduction in thymic T-cell production is thus valuable for seeking a potential direction of mechanistic studies regarding obesity and T-cell immunosenescence.

T cells, specifically αβT cells, develop in the thymus through rearrangement of T cell receptor gene segments. As a byproduct of the gene rearrangement, signal-joint T-cell receptor excision circles (sjTRECs, simply referred as TRECs in this study) are produced [12], [13], and these extrachromosomal DNA circles are not duplicated during mitosis. It is therefore possible to enumerate recent thymic emigrants (RTEs): They are newly produced naive T cells that recently emigrated from the thymus without having experienced cell division in the periphery, determined by quantifying TRECs. The TREC number in peripheral blood is thus an indicator of thymic capability to produce T cells [13], [14].

A long-term multidimensional research project in a fixed cohort of atomic-bomb survivors, the Adult Health Study (AHS) [15] at the Radiation Effects Research Foundation (RERF) makes it possible to comprehensively investigate immunological alterations related to various disease risk factors, such as aging and obesity, in addition to radiation exposure. In the present study, we evaluated the hypothesis that obesity contributes to human T-cell immunosenescence, by examining TREC numbers in peripheral blood among 1,073 AHS subjects. We found that lower TREC numbers were associated with higher age and were significantly associated with obesity indicators and related diseases among the study subjects, which supports our hypothesis.

## Materials and Methods

### Study Subjects

The AHS cohort was established in 1958, with enrollment of a total of 23,000 atomic-bomb survivors, and each participant received health examinations at the RERF every 2 years [15]. The population of this study was designed to examine immunological phenotypes in a subset of the AHS cohort, as previously reported [16]. A total of 1,073 atomic-bomb survivors who donated blood samples between 2003 and 2009 for immunological measurements was selected for this study, after excluding subjects diagnosed with rheumatism and autoimmune diseases which are known to influence TREC numbers in peripheral blood [17], [18].

### Ethics Statement

This study was approved by RERF’s institutional review committee, the Human Investigation Committee, and was conducted according to the principles expressed in the Declaration of Helsinki. All subjects gave written informed consent before each examination.

### Flow Cytometry Evaluation of Naive T Cell Populations

The percentages of naive CD4 and CD8 T-cell populations were measured using a FACScan (BD Biosciences) as described previously [19]. Briefly, the percentages of the T-cell populations in the lymphocyte fraction were analyzed using a combination of FITC-labeled anti-CD45RA (Coulter-Immunotech), PE-labeled anti-CD62L, and PerCP-labeled anti-CD4 or PerCP-labeled anti-CD8 monoclonal antibodies (BD Biosciences). CD45RA^+^/CD62L^+^ cells in CD4 and CD8 T-cell populations were identified as naive CD4 and CD8 T cells, respectively. Note that we used only the CD8-bright expression to identify CD8 T cells, in order to exclude NK cells which are dully CD8 positive.

### Isolation of CD4 and CD8 T-cell Populations

Mononuclear cell fractions in peripheral blood were separated by the Ficoll-Hypaque gradient technique. Approximately ten million mononuclear cells were stained with 10 μl of FITC-labeled CD4 antibody (BD Biosciences) and 25 μl PE-Cy5-labeled CD8 (BD-PharMingen, San Diego, CA, USA) in 200 μl PBS containing 1% FCS, for 30 min on ice. The cells were washed and resuspended in PBS containing 1% FCS and 0.1% sodium azide, and isolated by a FACS Vantage SE (BD Biosciences). Each CD4 and CD8 T-cell fraction was washed with PBS, and the cell pellets were stored at −20°C until used for TREC measurement.

### Measurement of TREC Numbers

TREC numbers in 1×10^5^ CD4 and CD8 T cell fractions were enumerated using the method reported by Yasunaga et al [20]. The procedure was modified to use crude DNA extracts resulting from a single treatment with proteinase K, which proved to be suitable for accurate quantification of the TRECs in preliminary experiments (data not shown). In brief, 7 μl (7 μg) of proteinase K (Sigma-Aldrich) was used per 10^5^ cell pellets, and the cells were digested at 56°C for 2 hours in the presence of 0.02% NP-40, 50 mM KCl, and 10 mM Tris-HCl (pH 8.4). They were then incubated at 95°C for 30 min. Sequences of primers and probes used for DNA amplifications were similar to those reported by Yasunaga et al [20]: Primers used for amplification of TREC were 5′-TCCCTTTCAACCATGCTGACA-3′ and 5′-TGCCTATGCATCACCGTGC-3′. The probe was 5′-CTCTGGTTTTTGTAAAGGTGCCCACTCCTG-3′ labeled with fluorescent FAM (reporter) at the 5′ end and fluorescent TAMRA (quencher) at the 3′ end. To measure cell equivalents in real-time PCR, the *recombination activating gene 1* (*RAG-1*) sequence in each sample was quantified by a method similar to that for TREC. The sequences of primers for *RAG-1* exon 2 detection were also similar to those reported by Yasunaga et al [20]: 5′-CCCACCTTGGGACTCAGTTCT-3′ and 5′-CACCCGGAACAGCTTAAATTTC-3′, and the probe was 5′-CCCCAGATGAAATTCAGCACCCACATA-3′ labeled with FAM at the 5′ end and TAMRA at the 3′ end. The crude DNA extracts (7 μl) were mixed with 10 μl of AbsoluteTM QPCR Mixes (ABgene House, Surrey, UK), and 1 μl each of the primer (final concentration, 0.3 μM) and probe (final concentration, 0.2 μM) were added to the mixture. The PCR conditions were 50 cycles of 15 seconds at 95°C followed by 60 seconds at 60°C. All experiments were performed and analyzed using a PRISM 7900 Sequence Detection System (Applied Biosystems, Foster City, CA). The number of TRECs in each sample was calculated using the following formula:
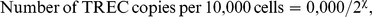
where χ = cycles required for significant amplification of TREC) − (cycles required for the significant amplification of *RAG-1*) –1.

### Information on Lifestyle/Environmental Factors and Clinical Data

Information on alcohol consumption and smoking at the time of TREC measurement was obtained from questionnaires at the AHS health examinations in 2003–2009. Clinical information such as BMI, total cholesterol, hemoglobin A1c (HbA1c) and diseases was also obtained at the AHS examinations along with medical chart review. Type-2 diabetes, fatty liver, hypertension and solid cancer were classified according to the International Classification of Diseases code as follows: diabetes (E11–E14), fatty liver (K70 and K76), hypertension (I10–I15), solid cancer (C00–C97, D00–D09). In addition, especially regarding BMI, the oldest data available for each subject (mostly over 40 years before TREC measurement, referred to as “past BMI”) were used in addition to data at the time of TREC measurement. Radiation exposure dose was estimated by the DS02 dosimetry system [21], based on the weighted lung dose computed as the γ dose plus 10 times the neutron dose.

### Data Analysis

A standard multiple linear regression method was used to determine the natural log-transformed number of TRECs (N = 1002 for CD4 TRECs, N = 952 for CD8 TRECs) by converted age at the time of examination = (age at examination (years old) −70)/10, gender (0 for male, 1 for female), radiation exposure dose in Gray, and obesity indicators: converted BMI = BMI (kg/m^2^) −22.7 (median), converted past BMI = past BMI (kg/m^2^) −20.8, converted total cholesterol = (total cholesterol (mg/dl) −206)/100, converted HbA1c = HbA1c (%) −5.4, converted CRP = CRP (mg/dl) −0.07, diabetes (1 if diagnosed, otherwise 0), fatty liver (1 or 0), hypertension (1 or 0). Alcohol consumption (converted to 10 grams of ethanol/day), smoking (10 cigarettes/day) and cancer (1 or 0) were also used for adjustments. Because the zero value was impossible for log transformation, the value showing zero for the number of TREC copies per 10,000 cells was replaced with 0.05 when the value was transformed into the natural log. In regression analyses, a forward stepwise procedure was also used for 8 obesity-related variables: BMI, past BMI, total cholesterol, HbA1c, CRP, diabetes, fatty liver, and hypertension. Four variables (past BMI, CRP, diabetes, and fatty liver) were consequently selected (significant level to select, p<0.2) to construct statistical models. Multivariate analysis, specifically generalized estimating equation (GEE) analysis, was performed to investigate whether there were differences in effects of age, gender, and radiation dose on TRECs between CD4 and CD8 T cells. The detailed methods of GEE are found in File S1. All statistical analyses were carried out using the STATA software (Stata/SE 9.2 for Windows, StataCorp LP, College Station, TX).

## Results

**Figure 1:**
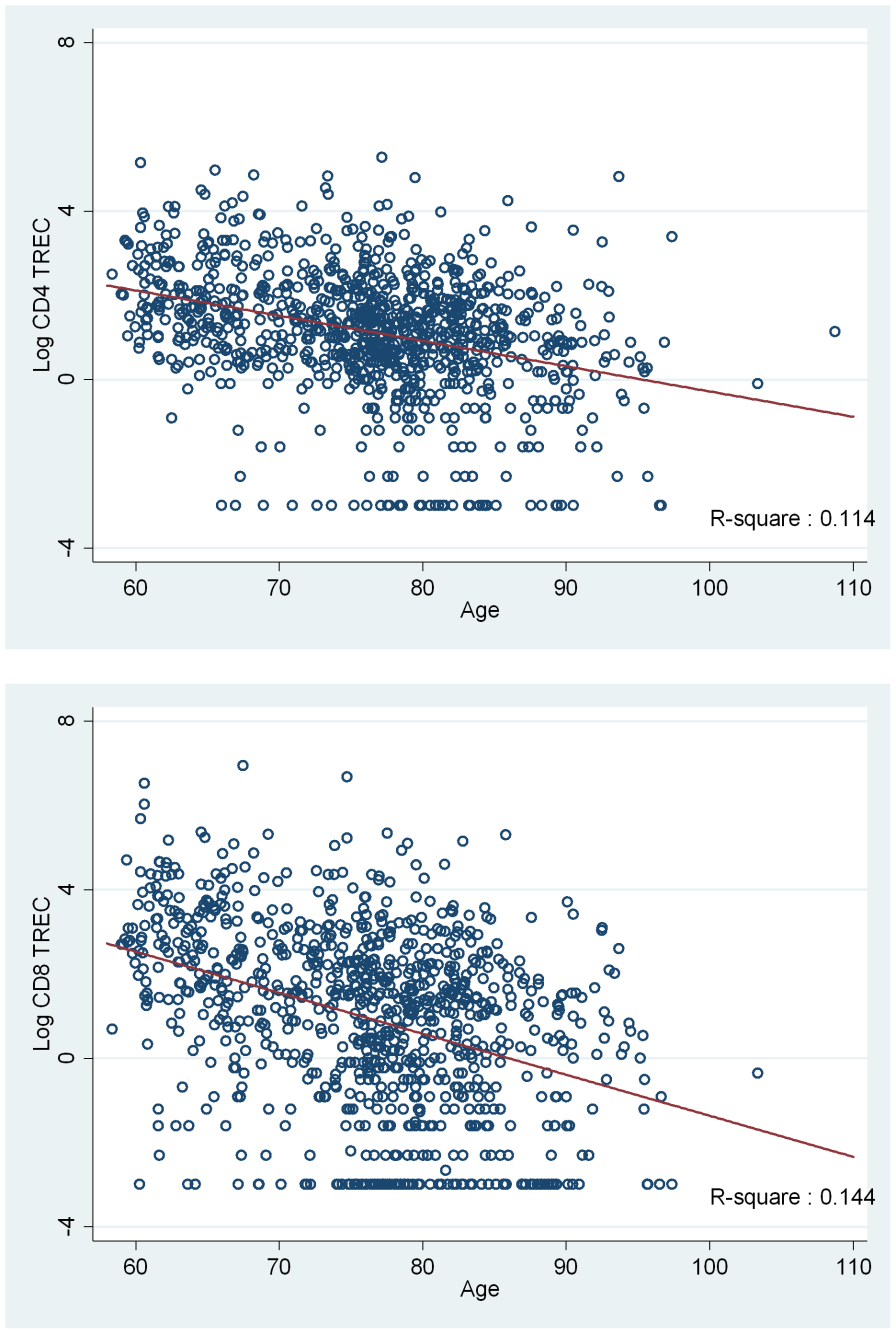
TREC numbers and age among aging atomic-bomb survivors. Scatter diagrams of age at the time of examination and CD4 TRECs or CD8 TRECs. Each dot represents a single subject. Regression lines and r-square values in simple linear regression are indicated. TREC stands for T-cell receptor excision circle.

The basic characteristics and obesity indicators of the study subjects are shown in Table 1. In addition, TREC numbers showed a strong positive correlation with naive T cell percentages in lymphocytes (Table S1 in File S1). To investigate the effects of age, gender, and radiation exposure on T-cell production, we first conducted multiple linear regression analyses of the number of TREC copies in the CD4 or CD8 T-cell fraction using age, gender, and radiation exposure as explanatory variables, along with alcohol consumption and smoking (Table 2). The results showed that TRECs in both CD4 and CD8 T cells were inversely associated with age at examination (p<0.001, also see Figure 1), and were higher among females than males (p<0.001), which was in accordance with previous studies [1], [13], [22]. However, no significant association with radiation dose was observed for either CD4 or CD8 TRECs.

**Table 1:** Study subjects (N = 1073).

Basic characteristics		Obesity indicators	
Age^a^	77.0 (61.8–90.0)	BMI (kg/m^2^)^a^	22.7 (17.5–28.6)
(at the time of examination)		BMI^b^	
		< = 19.5	166 (15.5)
Gender^b^		19.5–21.2	167 (15.6)
Male	378 (35.2)	21.2–22.9	191 (17.8)
Female	695 (64.8)	22.9–25.0	235 (21.9)
		>25.0	235 (21.9)
Radiation dose (Gray)^a^	0.166 (0–2.122)		
		Past BMI^a^	20.8 (17.3–26.2)
Alcohol (gram/day)^a^	0 (0–40)	Past BMI^b^	
Alcohol^b^		< = 19.5	318 (29.6)
0	651 (60.7)	19.5–21.2	291 (27.1)
0–20	279 (26.0)	21.2–22.9	216 (20.1)
20–40	64 (6.0)	22.9–25.0	157 (14.6)
40–60	32 (3.0)	>25.0	90 (8.4)
>60	14 (1.3)		
		Total cholesterol (mg/dl)^a^	206.0 (149.0–267.3)
Smoking (cigarette/day)^a^	0 (0–18.7)		
Smoking^b^		HbA1c (%)^a^	5.4 (4.8–7.4)
0	945 (88.1)		
0–20	113 (10.5)	CRP (mg/dl)^a^	0.070 (0.008–0.804)
>20	14 (1.3)		
		Diabetes cases^b^	223 (20.8)
CD4TREC^c^ (per 10^4^ cells)^a^	3.4 (0.2–29.6)	Fatty liver cases^b^	301 (28.1)
CD8TREC^c^ (per 10^4^ cells)^a^	3.6 (0.1–68.5)	Hypertension cases^b^	719 (67.0)

a Median (5–95% percentiles).

b Number (%).

c TREC stands for T-cell receptor excision circle.

**Table 2:** Regression analyses of TRECs.

Explanatory variables^a^	coefficient	p-value	Explanatory variables^b^	coefficient	p-value
Constant	1.33	<0.001	Constant	0.977	<0.001
Age	−0.671	<0.001	Age	−1.11	<0.001
Gender	0.393	<0.001	Gender	0.988	<0.001
Radiation dose	0.023	0.73	Radiation dose	0.044	0.64
Alcohol	−0.021	0.54	Alcohol	−0.021	0.67
Smoking	−0.077	0.33	Smoking	0.035	0.76

a Dependent variable: CD4 TRECs.

b Dependent variable: CD8 TRECs.

We additionally performed GEE analysis to determine if there were differences in the effects of age, gender, or radiation dose between CD4 and CD8 TRECs. The coefficient for interaction between age and “TREC2” (this variable represents the difference between the CD4 TREC and CD8 TREC expectations, where CD4 TREC is the basis) was a negative value (−0.439), which indicated that age-related decline in TRECs was significantly larger in CD8 T cells than in CD4 T cells (p<0.001, Table 3). In a similar way, gender difference (higher TRECs among females than males) was also significantly larger in CD8 T cells (p<0.001). The results are consistent with the notion that naive CD8 T cell numbers, compared with naive CD4 T cells, significantly decrease with age [23].

**Table 3:** GEE multivariate analysis.

Explanatory variables^a^	coefficient	p-value
Constant	0.958	<0.001
Age	−0.671	<0.001
Gender	0.397	<0.001
Radiation dose	0.023	0.70
Alcohol	−0.020	0.48
Smoking	−0.076	0.36
TREC2^b^	−0.907	<0.001
Age x TREC2	−0.439	<0.001
Gender x TREC2	0.605	<0.001
Radiation dose x TREC2	0.035	0.68
Alcohol x TREC2	0.006	0.89
Smoking x TREC2	0.110	0.29

a Dependent variable: the bivariate variable of standardized CD4 TRECs and CD8 TRECs.

b Effect of TREC2 represents the difference of the expectations of CD4 TRECs and CD8 TRECs, or the subtraction of the CD4 TREC expectation from the CD8 TREC expectation.

In order to investigate the relationship between obesity and TRECs, we then conducted regression analyses using a single obesity indicator as an explanatory variable in addition to age, gender, radiation dose, alcohol consumption, smoking, and cancer (because some types of cancer are known to be related to obesity). The results can be summarized as follows: lower CD4 TRECs were significantly associated with higher past BMI, HbA1c, CRP, or diagnosis with type-2 diabetes or fatty liver (Table 4). Very similar results were obtained for CD8 TRECs.

**Table 4:** Regression analyses of TRECs using a single obesity indicator.

Regression of CD4 TRECs^a^	coefficient	p-value	Regression of CD8 TRECs^a^	coefficient	p-value
BMI	−0.015	0.26	BMI	−0.033	0.086
Past BMI	−0.040	0.016	Past BMI	−0.039	0.10
Total cholesterol	−0.035	0.78	Total cholesterol	0.310	0.084
HbA1c	−0.131	0.010	HbA1c	−0.142	0.045
CRP	−0.227	0.032	CRP	−0.304	0.043
Diabetes	−0.349	0.001	Diabetes	−0.422	0.007
Fatty liver	−0.242	0.014	Fatty liver	−0.414	0.003
Hypertension	−0.088	0.35	Hypertension	−0.241	0.074

a Age, gender, radiation dose, alcohol consumption, smoking, and cancer were also adjusted in each regression analysis.

Finally, we conducted regression analyses simultaneously using multiple obesity indicators. We also applied a forward stepwise procedure for obesity indicators (significant level to select, p<0.2). HbA1c was not used simultaneously with diabetes because of the collinearity. Consequently, negative coefficients of CRP, diabetes, and fatty liver on TRECs were observed in both CD4 and CD8 T cells (Table 5). Similar results were obtained using the level of HbA1c instead of diagnosis with diabetes (Table S2 in File S1).

**Table 5:** Regression analyses of TRECs using multiple obesity indicators.

Regression of CD4 TRECs^a^	coefficient	p-value	Regression of CD8 TRECs^a^	coefficient	p-value
BMI	0.014	0.39	BMI	0.002	0.93
Past BMI	−0.041	0.040	Past BMI	−0.023	0.43
Total cholesterol	−0.129	0.31	Total cholesterol	0.149	0.42
CRP	−0.198	0.067	CRP	−0.221	0.15
Diabetes	−0.247	0.035	Diabetes	−0.282	0.097
Fatty liver	−0.174	0.11	Fatty liver	−0.377	0.015
Hypertension	−0.030	0.76	Hypertension	−0.162	0.25
**Regression of CD4 TRECs** a **^,^** b	**coefficient**	**p-value**	**Regression of CD8 TRECs** a **^,^** b	**coefficient**	**p-value**
Past BMI	−0.032	0.055	CRP	−0.251	0.094
CRP	−0.200	0.058	Diabetes	−0.319	0.047
Diabetes	−0.272	0.016	Fatty liver	−0.317	0.029
Fatty liver	−0.138	0.18			

a Age, gender, radiation dose, alcohol consumption, smoking, and cancer were also adjusted.

b A forward stepwise procedure was used for 7 obesity-related variables: BMI, past BMI, total cholesterol, CRP, diabetes, fatty liver, and hypertension. Four variables (past BMI, CRP, diabetes, and fatty liver) were consequently selected (significant level to select, p<0.2) to construct statistical models.

## Discussion

In the present study, we investigated the effects of aging, radiation exposure, and obesity on TREC numbers in peripheral blood among a subset of the atomic-bomb survivor study cohort, where the youngest individual was 58 years old. Previous studies have shown that aging has an impact on thymic capability to produce new T cells, by examining age-related changes in TREC numbers [1], [13], [22]. The present study indicated that TREC numbers continued to become lower after the age of 60, and that the age slope of TRECs appeared comparable to those reported for individuals aged 60 to 100 in other studies [9], [24].

In addition, we found that the negative effect of age on TRECs was greater in CD8 T cells than in CD4 T cells (Table 3), which is in agreement with the notion that naive CD8 T cells exhibit numerical attrition at more significant levels than naive CD4 T cells [23]. The differences between CD4 and CD8 T cells may partly be attributable to aging changes in the maturation process of single-positive (CD4^+^ or CD8^+^) thymocytes in the thymus [25], or the longer lifespan of naive CD4 T cells in the periphery [26]. Furthermore, the gender difference in TREC numbers―i.e., lower numbers among aged males than aged females―was also greater in CD8 T cells than in CD4 T cells (Table 3). It is thus likely that, compared with the human CD4 T-cell population, the human CD8 T-cell population is more likely to be affected by intrinsic factors such as aging and sex hormones: This may be explained by the difference in antigen recognition systems between CD4 and CD8 T cells: The former primarily recognize extracellular peptide antigens presented by MHC class II, and the latter recognize intracellular ones (including persistently infected microbes) presented by MHC class I.

Studies of atomic-bomb survivors have consistently found reduced numbers/percentages of peripheral naive CD4 and CD8 T cells, as well as impaired T cell functions, in a radiation dose-dependent manner [19], [27]. In the present study, however, we did not find any significant radiation effect on TRECs in either CD4 or CD8 T cells (Table 2). This may be because the radiation effect on peripheral TRECs might have been diluted since exposure to atomic-bomb radiation 60 years ago. Furthermore, the question remains whether exposure to higher-dose radiation would produce the same results, since median dose of the study population was relatively low, i.e., 166 mGray. To compensate for the limitations of this study, different study designs are required: analysis of biosamples immediately after radiation exposure, longitudinal analysis in a fixed cohort, and determination of radiation levels affecting TRECs using animal models.

There were significant inverse associations between TREC numbers and multiple obesity indicators among atomic-bomb survivors (Tables 4 and 5). Consistent with those findings, a previous study found a negative correlation between TRECs in peripheral blood mononuclear cells and BMI among dozens of individuals [5]. Technically, TREC numbers in peripheral T cells could be a measure of expansion of peripheral T cells and cell death of RTEs in addition to thymic output [13], since both intensive expansion of T cells and cell death of RTEs should decrease TREC levels. In fact, CD4 and CD8 T cell numbers were positively associated with some, but not all, obesity indicators in the study subjects (data not shown), suggesting that the T-cell expansion associated with obesity may also contribute to the reduction of TREC levels to some extent. However, regression analyses using absolute TREC numbers in 1 ml peripheral blood (not those in CD4 or CD8 T cells) still found inverse associations with most obesity indicators (Table S3 in File S1). On the other hand, a study of mice showed that obesity compromised T-cell production by increasing apoptosis of developing thymocytes in the thymus [5], but it is unlikely that RTEs are preferentially susceptible to cell death in obesity. Thus, it is likely that obesity is actually related to reduction in thymic T-cell production levels in humans, although T-cell expansion also seems to be a factor. Moreover, our analyses showed that BMI measured mostly 40 years ago was more closely associated with reduced CD4 TRECs than BMI measured at the time of TREC measurement (Table 5). This is not conclusive, but it may imply that obesity is not merely a related factor but a cause of accelerated T-cell aging in humans, as is the case with mice and non-human primates [5], [28], [29]. Our findings therefore support the hypothesis that obesity contributes to T-cell aging by reducing newly produced T cells in the human T-cell pool. Aiming to reinforce the hypothesis, we are currently studying the relationship between obesity and other T-cell aging markers, such as naive T-cell counts and T-cell telomere lengths.

Obesity, defined as excess adiposity with high BMI, is a multisystem disorder (2), and it is now obvious that obesity is a factor in increased mortality from type-2 diabetes, cardiovascular disease, and cancer [30]. There is also evidence that obese individuals are more susceptible to various types of infections, including postoperative and nosocomial infections [31], [32], and development of these various age-related diseases and symptoms is often related to attenuated immunity as well as chronic inflammatory status [33], [34]. Most recently, a human population study showed that thymic dysfunction based on TRECs was an independent predictor of all-cause mortality in the elderly [35]. It is thus plausible that attenuated immune competence due to reduced thymic T-cell production is a possible mechanistic link between obesity and disease development in the elderly.

The precise mechanisms that can explain how obesity compromises the thymic T-cell production are elusive, but considering the broad effects of obesity, it is plausible that the mechanisms involved are complex and multifactorial [2]. One potential mediator in coupling obesity to T-cell immunity is the mechanistic target of rapamycin (mTOR) signaling pathway: The mTOR complex is a signal integrator for nutrients, growth factors, and intracellular energy levels, and the chronic activation of mTOR signaling during obesity promotes lipid synthesis and adipocyte differentiation [36]. In addition to such a potential effect on the lymphoid microenvironment, one recent report suggests that mTOR activation is linked to age-related changes in hematopoietic stem cells [37]. Leptin, ghrelin, and insulin-like growth factor-1 are also known to be important mediators in acceleration of T-cell immunosenescence by obesity [2], [38]. Compromised T-cell immunity may, in turn, contribute to metabolic syndrome, implying a bidirectional interaction between obesity and T-cell immunity [39], [40]. Further exploration of such an interaction could offer novel approaches to suppressing the development of age-related diseases by enhancing immune competence.

## Supporting Information

File S1 GEE analysis methods and Tables S1–S3. File S1 shows detailed methods of generalized estimating equation (GEE) analysis, correlation between TRECs and naive T cell percentages (Table S1), regression analyses of TRECs using HbA1c (Table S2), and regression analyses of absolute TREC numbers using an obesity indicator (Table S3).
(DOCX)
