# Correction: Inverse Associations between Obesity Indicators and Thymic T-Cell Production Levels in Aging Atomic-Bomb Survivors

**DOI:** 10.1371/journal.pone.0101097

**Published:** 2014-06-19

**Authors:** The PLOS ONE Staff

In the Materials and Methods section under “Measurement of TREC Numbers,” the formula for calculating the number of TRECs per sample is incorrect. The correct formula is: Number of TREC copies per 10,000 cells  =  10,000/2^χ^
