# Supplementary material for: Inverse Associations between Obesity Indicators and Thymic T-Cell Production Levels in Aging Atomic-Bomb Survivors
Source: PLoS One. 2014 Mar 20;9(3):e91985. doi: 10.1371/journal.pone.0091985 (PMC3961282; doi:10.1371/journal.pone.0091985)
Supplement: File S1 — GEE analysis methods and Tables S1–S3. File S1 shows detailed methods of generalized estimating equation (GEE) analysis, correlation between TRECs and naive T cell percentages (Table S1), regression analyses of TRECs using HbA1c (Table S2), and regression analyses of absolute TREC numbers using an obesity indicator (Table S3). (DOCX) [file pone.0091985.s001.docx]

**Inverse associations between obesity indicators and thymic T-cell production levels in aging atomic-bomb survivors**

Kengo Yoshida^*^, Eiji Nakashima, Yoshiko Kubo, Mika Yamaoka, Junko Kajimura, Seishi Kyoizumi, Tomonori Hayashi, Waka Ohishi, and Yoichiro Kusunoki

^*^Correspondence should be addressed to K. Yoshida (kyoshi@rerf.or.jp)

Department of Radiobiology/Molecular Epidemiology, Radiation Effects Research Foundation, 5-2 Hijiyama Park, Minami Ward, Hiroshima 732-0815, Japan

Tel: +81 82 261 3131

Fax: +81 82 261 3170

**Detailed methods of generalized estimating equation (GEE) analysis**

Bivariate responses, the natural logs of CD4 TREC and CD8 TREC, are assumed to be normally distributed with heteroscedastic variances. The scaling for these two response variables was performed by division by the square roots of the residual sum of squares from two multiple regressions. To obtain those values, two multiple linear regressions for the normal responses Y_j_, natural logs of CD4 TREC for j=1 and of CD8 TREC for j=2, were made,

1. Y_j_ = 1 + gender + age + dose + smoking + alcohol + err_j_,

where 1 stands for intercept, and err_j_ (j=1, 2) are normally distributed with zero means and different variances. The square roots of the residual sum of squares for these two responses are denoted as H_j_ (j=1, 2) with H_1_ = 1.3787 (N = 1,002) and H_2_ = 1.9191 (N = 952). We transformed Y_j_ to Z_j_ = Y_j_/H_j_ and also transformed the variables X_k_ in the regression of Z_j_ to W_jk_ = X_k_/H_j_ for j=1, 2, where X_k_ is gender for k=1, age for k=2, dose for k=3, smoking for k=4 and alcohol for k=5.

We used the generalized estimating equation (GEE) for bivariate responses with equal scales, GEE1. Due to the heteroscedasticity of the responses, we could not apply the bivariate GEE1 to Y_j_ (j=1, 2): We instead applied bivariate GEE1 to Z_j_ for j=1, 2, where bivariate normal responses, Z_j_ (j=1, 2), have a similar variance approximately equal to one, and have a correlation. The GEE1 model for Z_j_ (j=1, 2) is,

(2) Z_j_ = 1 + W_j1_ + W_j2_ + W_j3_ + W_j4_ + W_j5_ + D(j) + I(j=2)*W_j1_

+ I(j=2)* W_j2_ + I(j=2)*W_j3_ + I(j=2)*W_j4_ + I(j=2)*W_j5_ + ERR_j_,

where I(j=2) is indicator variable, i.e., I(j=2)=0 if j=1 and I(j=2)=1 if j=2; D(j)=(j-1)/SD_j_ stands for the difference between the intercepts of two responses of Z_1_ and Z_2_, I(j=2)*W_jk_ is the interaction term between indicator variable I(j=2) and variable W_jk_, and ERR_j_ for j=1, 2 represent the correlated bivariate normal errors with a 2 by 2 covariance matrix, each diagonal element being approximately equal to one. The estimated correlation using GEE1 was 0.438.

The parameter estimates from GEE1 for Z_j_ (j=1, 2) and from GEE1 for Y_j_ (j=1, 2) are both consistent with the true parameters. In our setting, the application of the former GEE1 for Z_j_ (j=1, 2) is correct for homoscedastic working variances, and the application of the latter GEE1 for Y_j_ (j=1, 2) is incorrect for homoscedastic working variances. Due to the correct specification of working variances, the GEE1 for Z_j_ (j=1, 2) gives more efficient estimates with smaller standard errors than the GEE1 for Y_j_ (j=1, 2). In addition, the GEE1 estimates with Z_j_ (j=1, 2) are highly efficient (Liang, K. Y and Zeger, S. L. Longitudinal data analysis using generalized linear models. *Biometrika* 1986;73: 13-22). The Wald tests for the significance of the parameters were made using robust standard errors derived from GEE1 for the bivariate responses Z_j_ (j=1, 2).

| Table S1. Correlation between TRECs and naive T cell percentages in lymphocytes | | | |
| --- | --- | --- | --- |
|  |  |  |  |
|  |  | CD4 naive T-cells |  |
| CD4 TREC | correlation coefficient^a^ | 0.26 |  |
|  | p-value | < 0.00001 |  |
|  |  |  |  |
|  |  | CD8 naive T-cells |  |
| CD8 TREC | correlation coefficient^a^ | 0.38 |  |
|  | p-value | < 0.00001 |  |
|  |  |  |  |
| ^a^Adjusted for age, gender, radiation dose, alcohol consumption, and smoking. | | |  |

| Table S2. Regression analyses of TRECs using HbA1c | | |
| --- | --- | --- |
|  |  |  |
| Regression of CD4 TRECs^a^ | coefficient | p-value |
| BMI | 0.016 | 0.33 |
| Past BMI | -0.043 | 0.035 |
| Total cholesterol | -0.117 | 0.36 |
| HbA1c | -0.077 | 0.16 |
| CRP | -0.205 | 0.062 |
| Fatty liver | -0.188 | 0.086 |
| Hypertension | -0.026 | 0.80 |
|  |  |  |
| Regression of CD4 TRECs^a, b^ | coefficient | p-value |
| Past BMI | -0.033 | 0.054 |
| HbA1c | -0.087 | 0.10 |
| CRP | -0.205 | 0.056 |
| Fatty liver | -0.149 | 0.15 |

| Regression of CD8 TRECs^a^ | coefficient | p-value |
| --- | --- | --- |
| BMI | 0.006 | 0.80 |
| Past BMI | -0.019 | 0.50 |
| Total cholesterol | 0.180 | 0.33 |
| HbA1c | -0.085 | 0.27 |
| CRP | -0.203 | 0.19 |
| Fatty liver | -0.411 | 0.009 |
| Hypertension | -0.150 | 0.29 |
|  |  |  |
| Regression of CD8 TRECs^a, b^ | coefficient | p-value |
| CRP | -0.260 | 0.084 |
| Fatty liver | -0.388 | 0.006 |

| ^a^Age, gender, radiation dose, alcohol consumption, smoking, and cancer were also adjusted. |
| --- |
| ^b^A forward stepwise procedure was used for 7 obesity-related variables: BMI, past BMI, total cholesterol, HbA1c, CRP, fatty liver, and hypertension. Four variables (past BMI, HbA1c, CRP, and fatty liver) were consequently selected (significant level to select, p < 0.2) to construct statistical models. |

| Table S3. Regression analyses of absolute TREC numbers using an obesity indicator | | |
| --- | --- | --- |
|  |  |  |
| Regression of CD4 TRECs^a^ | coefficient | p-value |
| BMI | 0.004 | 0.79 |
| Past BMI | -0.038 | 0.040 |
| Total cholesterol | 0.058 | 0.67 |
| HbA1c | -0.114 | 0.043 |
| CRP | -0.251 | 0.029 |
| Diabetes | -0.355 | 0.003 |
| Fatty liver | -0.182 | 0.091 |
| Hypertension | 0.003 | 0.97 |

| Regression of CD8 TRECs^a^ | coefficient | p-value |
| --- | --- | --- |
| BMI | -0.017 | 0.39 |
| Past BMI | -0.038 | 0.14 |
| Total cholesterol | 0.324 | 0.089 |
| HbA1c | -0.103 | 0.18 |
| CRP | -0.265 | 0.094 |
| Diabetes | -0.356 | 0.031 |
| Fatty liver | -0.401 | 0.007 |
| Hypertension | -0.221 | 0.124 |

^a^Age, gender, radiation dose, alcohol consumption, smoking, and cancer were also adjusted in each regression analysis.
